# Comparative review of outcomes: single-incision laparoscopic total extra-peritoneal sub-lay (SIL-TES) mesh repair versus laparoscopic intraperitoneal onlay mesh (IPOM) repair for ventral hernia

**DOI:** 10.1007/s13304-022-01288-4

**Published:** 2022-04-15

**Authors:** Tingfeng Wang, Rui Tang, Xiangzhen Meng, Yizhong Zhang, Liangliang Huang, Aili Zhang, Weidong Wu

**Affiliations:** 1 Department of General Surgery, Shanghai Pudong Hospital, Fudan University Pudong Medical Center, 2800 Gongwei Road, Pudong, Shanghai, 201399 China; 2 Department of Hernia and Abdominal Wall Surgery, Shanghai East Hospital, Tongji University, 150 Jimo Road, Pudong, Shanghai, 200120 China; 3 Department of General Surgery, China Medical University Affiliated Shengjing Hospital, 36 Sanhao Road, Shenyang, 110004 China; 4 Department of General Surgery, Affiliated Hospital of Medical School of Ningbo University, 247 Renmin Road, Ningbo, 31500 China; 5 Department of Operation Room, Shanghai Medical College of Fudan University Affiliated Huadong Hospital, 221 Yanan Xi Road, Shanghai, 200041 China; 6 Department of Gastrointestinal Surgery, Shanghai Jiaotong University Affiliated First People’s Hospital, Shanghai General Hospital, 85 Wujin Road, Shanghai, 200080 China

**Keywords:** SIL-TES, IPOM, Ventral hernia, Quality of life, Cost-effective analysis

## Abstract

To compare outcomes between single-incision laparoscopic totally extra-peritoneal sub-lay (SIL-TES) mesh repair and laparoscopic intraperitoneal onlay mesh (IPOM) repair of ventral hernia (VH). A retrospective selection of 104 patients who underwent VH repair (50 and 54 in the SIL-TES and IPOM groups, respectively) was made. Patient data were collected, and quality of life was evaluated using Carolinas Comfort Scale (CCS) 1 month and 3 months after surgery. There were no significant differences in sex, American Society of Anesthesiologists class, defect size, mesh area, estimated blood loss, and complication rate between the groups. Age was lower, body mass index was higher, prevalence of primary VH was significantly higher (*p* < 0.0001), and pain was less at 24 and 48 h post procedure (*p* < 0.0001) in the SIL-TES group. Drainage placement was more (*p* < 0.0001), operation time was shorter (*p* = 0.012), and hospitalization duration and total hospitalization cost were greater in the IPOM group than that in SIL-TES group (8.3 ± 0.3 vs 4.3 ± 0.4 days, *p* < 0.0001; $7126.9 ± 141.4 vs $2937.3 ± 58.3, *p* < 0.0001, respectively). Pain and movement limitation scores evaluated by CCS were significantly worse at 1 month (4.93 ± 0.28 vs 1.75 ± 0.28: *p* < 0.0001; 2.52 ± 0.24 vs 1.15 ± 0.18: p < 0.0001, respectively) and 3 months (4.32 ± 0.37 vs 0.9 ± 0.29: *p* < 0.0001; 2.06 ± 0.25 vs 0.69 ± 0.11: *p* < 0.0001, respectively) in IPOM group, compared with the according scores in SIL-TES group. There was no readmission within 30 days and no hernia recurrence at mean follow-up of 12 months. SIL-TES mesh repair is safe and effective and is superior to IPOM repair.

## Introduction

Laparoscopic intraperitoneal onlay mesh (IPOM) repair, first described by LeBlanc and Booth [1], is now considered a standard surgical procedure for ventral hernia (VH). Compared to the open approach, IPOM has obvious advantages, such as low wound complication rates and fast recovery. However, it also has some limitations. For example, it is associated with rare but serious complications that may follow intraperitoneal mesh placement, including visceral damage, ileus, mesh migration or mesh erosion, and enter cutaneous fistula caused by direct contact between the mesh and intraperitoneal viscera [2].

Therefore, it was necessary to find an alternative technique for VH repair. Many scholars tried to apply the transabdominal pre-peritoneal (TAPP) elements, and the total extra-peritoneal (TEP) approaches of inguinal hernia repair to VH repair. This resulted in the laparoscopic retro-muscular and pre-peritoneal procedure, and the first report of this procedure is found in a paper by Miserez published in 2002 [3]. Miserez described direct access to the retro-muscular plane in a small cohort of 15 patients, referring to the procedure as “endoscopic total pre-peritoneal” repair. After that, many articles on this new technique originating from different countries appeared. Bittner applied the “mini and less open sub-lay” technique to endoscopic repair and named it eMILOS [4]. Belyansky [5] adapted his enhanced-view TEP technique, originally used to treat complex inguinal hernias, for VH repair. Similarly, several other scholars reported cases of their patients who underwent totally endoscopic sub-lay repair [6–8]. Other techniques for VH repair, such as the expanded TAPP approach [9] and pre-peritoneal onlay mesh approach [10], have also been subsequently described. Unfortunately, it is challenging to separate and close the thin peritoneal flap with these approaches without robotic assistance. We began to realize the clinical value and prospects of this technique in China, and in 2016, we named it endoscopic sub-lay repair (ESR) [11]. ESR combines the total extra-peritoneal sub-lay (TES) approach and the transabdominal sub-lay (TAS) approach.

At the same time, single-incision laparoscopic surgery (SILS) drew increasing attention due to emphasis on postoperative pain, cosmetic results, and concerns regarding port-site incisional hernia. With professional single-access devices, such as LAGIS and Senscure, the length of incision (LoI) can be limited to 2.0–2.5 cm, resulting in low trocar-site hernia rates, significantly lower postoperative pain, and satisfactory results cosmetic effect [12–14]. Hence, SILS has become a commonly performed procedure in clinical practice.

This study compared clinical outcomes and quality of life (QoL) after VH repair between a new combination of TES and SILS (denoted as SIL-TES mesh repair) and the traditional IPOM repair.

## Methods

### Study design

A retrospective study of 50 patients who underwent elective SIL-TES mesh repair (the SIL-TES group) and 54 patients who underwent elective conventional IPOM repair (the IPOM group) for VH between October 2018 and October 2020 was conducted. The study included the following five hernia centers: Shanghai General Hospital, Shanghai (*n* = 15); China Medical University Affiliated Shengjing Hospital, Shenyang (*n* = 13); Affiliated Hospital of Medical School of Ningbo University, Ningbo (*n* = 12); Tongji University Affiliated Dongfang Hospital, Shanghai (*n* = 54); and Fudan University Pudong Medical Center, Shanghai (*n* = 10).

Basic patient characteristics obtained included age, gender, body mass index (BMI), American Society of Anesthesiologists (ASA) class, and hernia characteristics. Perioperative data included LoI, hernia defect area, area of mesh used, manner of mesh fixation, surgical time, estimated blood loss volume, rate of drainage placement, and rate of complications. Post-operative data included pain score, evaluated using the visual analog scale, complication rates (e.g., surgical-site infection, mesh infection, hematoma, and intestinal leak/fistula), hospital length of stay (LoS), hospitalization costs, readmission rate, and recurrence rate.

QoL assessments were performed in-person, by telephone, or by electronic communication between the clinical team and patients who consented to the study and data collection. QoL was assessed using the Carolina Comfort Scale (CCS) at one month and three months after the operation. CCS is a validated hernia-specific questionnaire with a 0–5 scale (0 indicating “no symptoms” and 5 indicating “disabling symptoms”) used to evaluate pain, mesh sensation, and movement limitation.

### Patient selection

The standardized preoperative workup of patients started with detailed history-taking and physical examination. All patients underwent routine computed tomography of the abdomen and pelvis for preoperative hernia measurement and operative planning. The study enrolled patients who underwent SIL-TES mesh repair or IPOM repair, aged 18–80 years, of preoperative ASA class 1 or class 2, with defect length < 4 cm, and with hernia located in M1–5 and L3–4 according to the European Hernia Society classification [15].

### Operative technique

#### SIL-TES mesh repair

**Fig. 1:**
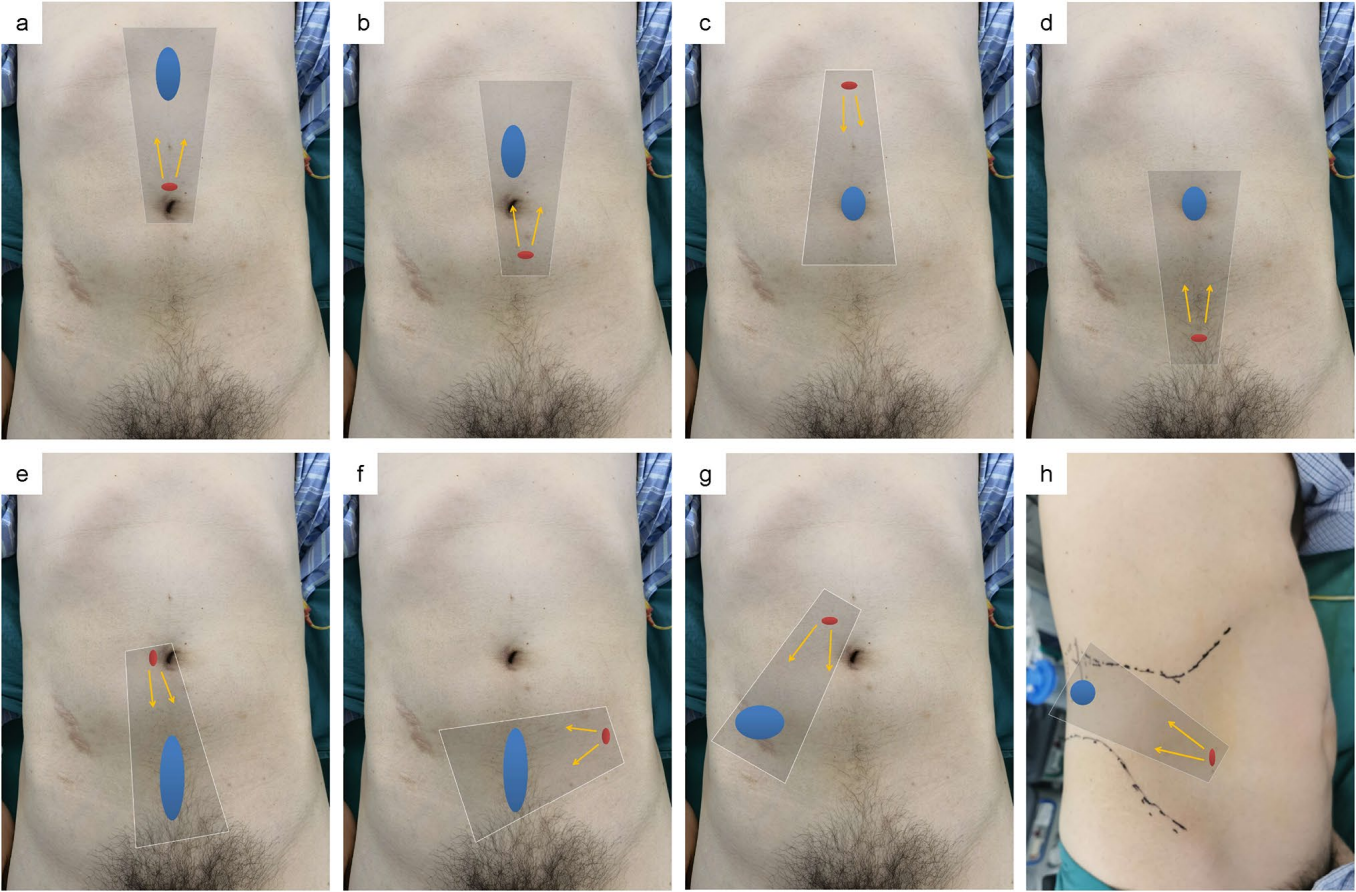
The typical incision layout in SIL-TES for defects in different regions. **a** M1; **b** M2; **c**, **d** M3; **e**, **f** M4–M5; **g** L2 and/or L3; **h** L4; gray shadow: Camera scope direction; red dot: Incision site; blue area: defect site; region L, M is based on the incisional hernia classification of EHS [15]

A port-site incision was made according to the location of the hernia defect, as shown in Fig. 1, and the optimal LoI was 2.0–2.5 cm. The skin, subcutaneous tissue, and anterior rectus sheath were cut in turn, and the rectus abdominis was then separated to install the port. In complex cases (e.g., hernia sac with dense attachments or large defects), a 5-mm auxiliary operating channel was optionally placed near the outer edge of the contralateral rectus abdominis.

**Fig. 2:**
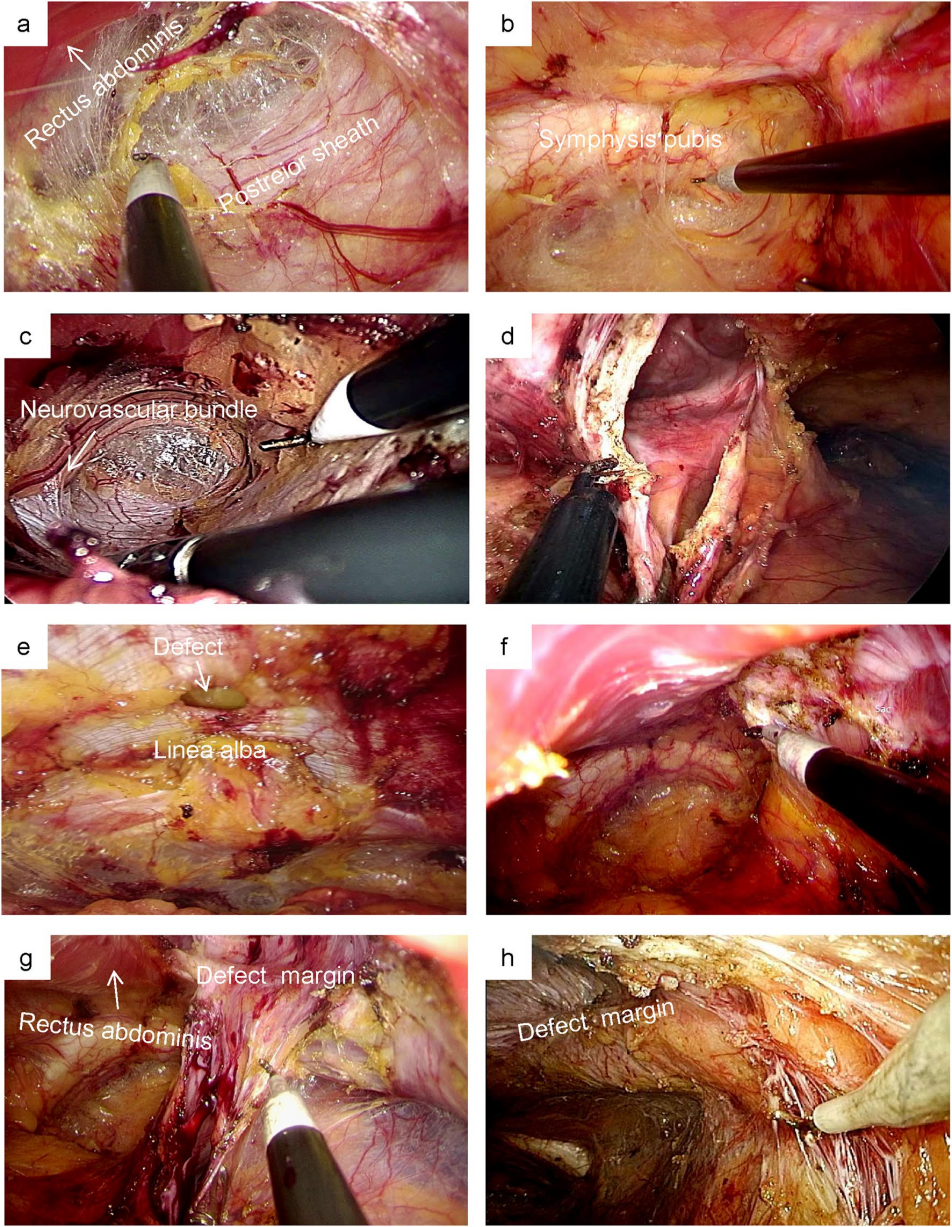

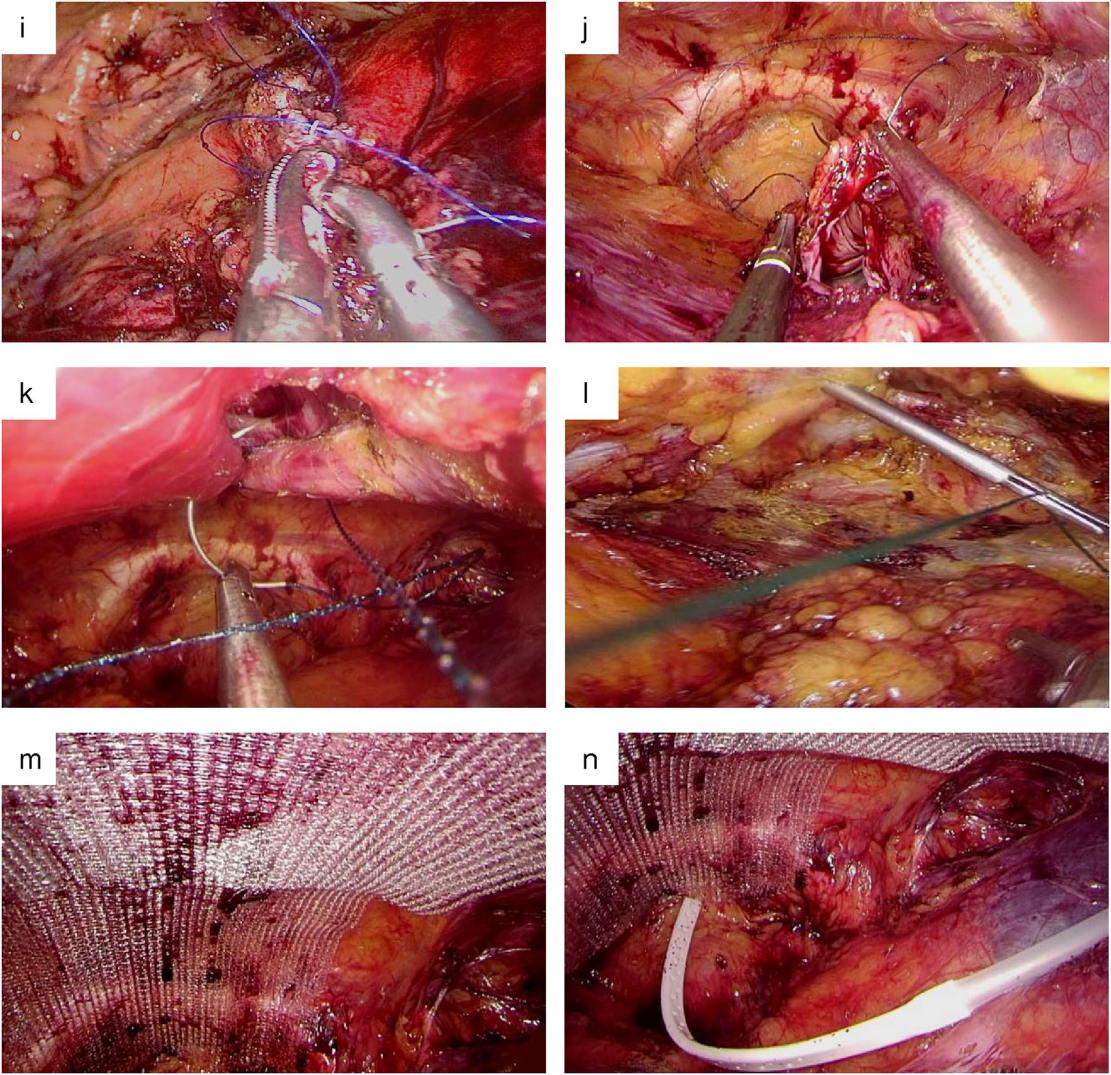
SIL-TES procedure. Separate in retro-rectus space (**a**) and Retzius space (**b**). **c** Expose and protect the neurovascular bundle. **d** Incise the medial aspect of the posterior rectus sheath. **e** linea alba hernia. **f** and **g** Incise the hernia sac. **h** Incise the lateral aspect of the posterior rectus sheath. Close posterior rectus sheath (**i**) and peritoneal laceration (**j**). Close hernia defect by continuous reverse sewing (k) or by intermittent trans-fascial sutures (**l**). Place the mesh (**m**) and drainage (**n**)

For lower midline and peripheral defects, the retro-muscular space was sharply dissected using electrocautery under laparoscopic vision (Fig. 2a). The dissection was further performed into the Retzius and Bogros spaces (Fig. 2b) until the hernia sac was encountered. For upper midline defects, we first expanded the retro-rectus space further in the cephalad direction. Bilateral posterior rectus sheaths were identified and released. It is critical to avoid injuring vascular nerves inside the semilunar line (Fig. 2c). The linea alba and umbilical ring are barriers to retro-rectus space expansion; therefore, it was necessary to incise the medial aspect of the posterior rectus sheath just superficial to the falciform ligament (Fig. 2d).

In small-sized incisional or primary hernias, we usually sharply dissected the distal attachments of the sac and mobilized it downward (Fig. 2e). Alternatively, the sac can be incised directly if it is difficult to free it (Fig. 2f and g). As all the lateral hernias in the SIL-TES group were categorized as L3 or L4, it was necessary to incise the lateral aspect of the posterior rectus sheath (Fig. 2h) and expand the upper part of the Bogros space without using the transversus abdominis release technique. After that, we proceeded with retro-rectus dissection in the cephalad direction.

The incised medial aspect of the posterior rectus sheath and peritoneal laceration caused by separation or active cutting can be closed under laparoscopic vision using barbed sutures in a running fashion (Fig. 2i and j). Alternatively, the latter can be brought out through the port and sutured under direct vision. Regarding hernia defect closure, continuous reverse sewing using barbed sutures and intermittent transabdominal stitches can be used, as shown in Fig. 2k and l.

Finally, a lightweight polypropylene mesh of dimensions 15 cm × 15 cm was tailored and placed in the retro-rectus space with a minimum of 5 cm of overlap over the defect in each direction, usually without fixation or with self-fixation (Fig. 2m). As shown in Fig. 2n, a drainage tube can be passed through the auxiliary operating channel in large incisional hernias. Still, in cases of small-sized and primary abdominal wall hernias, a drainage tube is not required. Pneumoperitoneum is released under direct vision after ensuring that the mesh is flat on the surface of the posterior rectus sheath. 

#### IPOM repair

Patients in the IPOM group underwent conventional laparoscopic IPOM repair. A Veress needle was used to create a pneumoperitoneum with a pressure of 14 mmHg, and three trocars were subsequently placed. The trocars were used for adhesiolysis, defect closure, and initial mesh positioning. Two additional contralateral trocars are typically placed to facilitate tacking the ipsilateral side of the mesh. Adhesiolysis was performed using cold scissors and limited advanced bipolar energy, and all the sac contents were reduced. Defect closure was performed using trans-fascial sutures. Intraperitoneal mesh reinforcement was aimed at providing 3–5 cm of overlap after defect closure. Thus, we mostly used mesh with a width of 15 cm. Circumferential fixation using absorbable tacks was followed with four to five transabdominal fixation stitches using #1 polypropylene suture. Drainage should be placed if extensive adhesiolysis was required or if an intestinal injury occurred during surgery.

### Statistical analysis

Data analysis was performed using SPSS 22.0 (IBM Corporation, Armonk, NY, USA). Quantitative variables were presented as mean ± standard error of the mean. Shapiro–Wilk test was used to test the normality of data. Quantitative variables were compared between the groups using the Student’s *t* test or Wilcoxon test. The Chi-square test or Fisher’s exact test compared qualitative variables. *p* values less than 0.05 were considered statistically significant.

## Results

The study included 104 patients: 50 patients in the SIL-TES group and 54 patients in the IPOM group. There were no significant differences in sex and ASA class between the two groups. However, BMI was significantly higher in the SIL-TES group than in the IPOM group (*p* < 0.0001), and age was significantly lower in the SIL-TES group than in the IPOM group (*p* = 0.0207). In addition, the prevalence of VH was significantly higher in the SIL-TES group (76%) than in the IPOM group. In comparison, the prevalence of incisional hernia was higher in the IPOM group (74%) than in the SIL-TES group (*p* < 0.0001). Umbilical hernia accounted for 55% of primary VHs in the SIL-TES group. Table 1 summarizes the basic patient characteristics.

**Table 1:** Basic patient characteristics

Variable	SIL-TES approach	IPOM approach	*p* value
*N*	50	54	
Age	57.0 ± 2.4	66.6 ± 1.6	0.0207
Body mass index (kg/m^2^)	27.5 ± 0.6	24.6 ± 0.4	< 0.0001
ASA(I/II)	26/14	23/21	0.2373
Gender (male/female)	18/22	16/28	0.4206
Type of VH			< 0.0001
Primary ventral hernia	38	14	
Umbilical hernia	21	12	
Spigelian hernia	7	0	
Linea Alba hernia	7	2	
Lumbar hernia	3	0	
Incisional hernia	12	40	
Concomitant defect			
Inguinal hernia	6	2	
Rectus abdominis diastasis	2	1	
Defect region			0.829
Midline	36	40	
Lateral	14	14	

Perioperative data, including LoI, hernia defect area, mesh area, manner of mesh fixation, surgical time, estimated blood loss volume, drainage placement rate, and intestinal injury rate were analyzed between the two groups. Hernia defect area and mesh area were slightly less in the SIL-TES group than in the IPOM group (14.6 ± 1.2 cm^2^ versus 16.8 ± 1.3 cm^2^ and 193.1 ± 10.4 cm^2^ versus 204.9 ± 9.7 cm^2^, respectively). Still, there were no statistically significant differences in these variables between the two groups. Further, 80% of patients in the SIL-TES group used intermittent transabdominal stitches to close the defect, less than those in the IPOM group (*p* < 0.0001). There was no difference in estimated blood loss volume between the two groups. However, operation time was significantly shorter in the IPOM group than in the SIL-TES group (115.6 ± 6.1 min versus 145.5 ± 10.4 min, *p* = 0.012). Intraoperative bowel injury occurred in two patients in the IPOM group, but it did not occur in the SIL-TES group. Drainage was placed significantly higher in the IPOM group than in the SIL-TES group (61% versus 20%, *p* < 0.0001). In the SIL-TES group, the mean LoI was 2.2 ± 0.4 cm, SILS was successfully performed in 90% of the operations, and no additional auxiliary operating channels were needed. Furthermore, tacks were not needed for mesh fixation in the SIL-TES group, but they were required for mesh fixation in all the patients in the IPOM group. In the SIL-TES group, mesh fixation was not necessary for 28% of patients, and at 28%, self-fixation was the most used mesh fixation method in the SIL-TES group. Table 2 summarizes the perioperative data.

**Table 2:** Perioperative data

Variable	SIL-TES approach	IPOM approach	*p* value
Mean LoI (cm)	2.2 ± 0.1	–	
Single incision (SI)	45 (90%)	0	
SI plus one	5 (10%)	0	
Mean defect area (cm^2^)	14.6 ± 1.2	16.8 ± 1.3	0.2518
Mean mesh area (cm^2^)	193.1 ± 10.4	204.9 ± 9.7	0.5648
Hernia defect closure			< 0.0001
Transabdominal stitches	40	54	
Barbed sutures	10	0	
Mesh fixation			
None	14 (28%)	0	
Self-fixing	14(28%)	0	
Suspension	6 (12%)	8(14.8%)	
Suture	3 (6%)	0	
Self-fixing and suspension	13 (26%)	0	
Tack	0	54(100%)	
Intestinal injury	0	2	
Mean estimated blood loss (mL)	12.6 ± 1.9	11.8 ± 1.5	0.8824
Mean surgical time (mins)	145.5 ± 10.4	115.6 ± 6.1	0.012
Drainage	10 (20%)	33 (61%)	< 0.0001

Mesh infection, hematoma, or intestinal leak/fistula did not occur in any patient in any group. However, there was one case of surgical-site infection in the SIL-TES group. One patient in the IPOM group developed intestinal obstruction after surgery and recovered after conservative treatment. In addition, hospital LoS was significantly longer in the IPOM group than in the SIL-TES group (8.3 ± 0.3 days versus 4.3 ± 0.4 days, *p* < 0.0001). Total hospitalization cost was also significantly higher in the IPOM group than in the SIL-TES group ($7126.9 ± 141.4 versus $2937.3 ± 58.3, *p* < 0.0001). There were no readmissions within 30 days and no hernia recurrences over a mean follow-up duration of 12 months. Table 3 presents the postoperative data.

**Table 3:** Post-operative data

Variable	SIL-TES approach	IPOM approach	*p* value
SSI	1	0	
Mesh infection	0	0	
Hematoma	0	0	
Intestinal obstruction	0	1	
Intestinal leak/fistula	0	0	
Mean LoS (days)	4.3 ± 0.4	8.3 ± 0.3	< 0.0001
Hospitalization costs (USD)	2937.3 ± 58.3	7126.9 ± 141.4	< 0.0001
Readmission	0	0	
Recurrence	0	0	
Incision hernia	0	0	

**Fig. 3:**
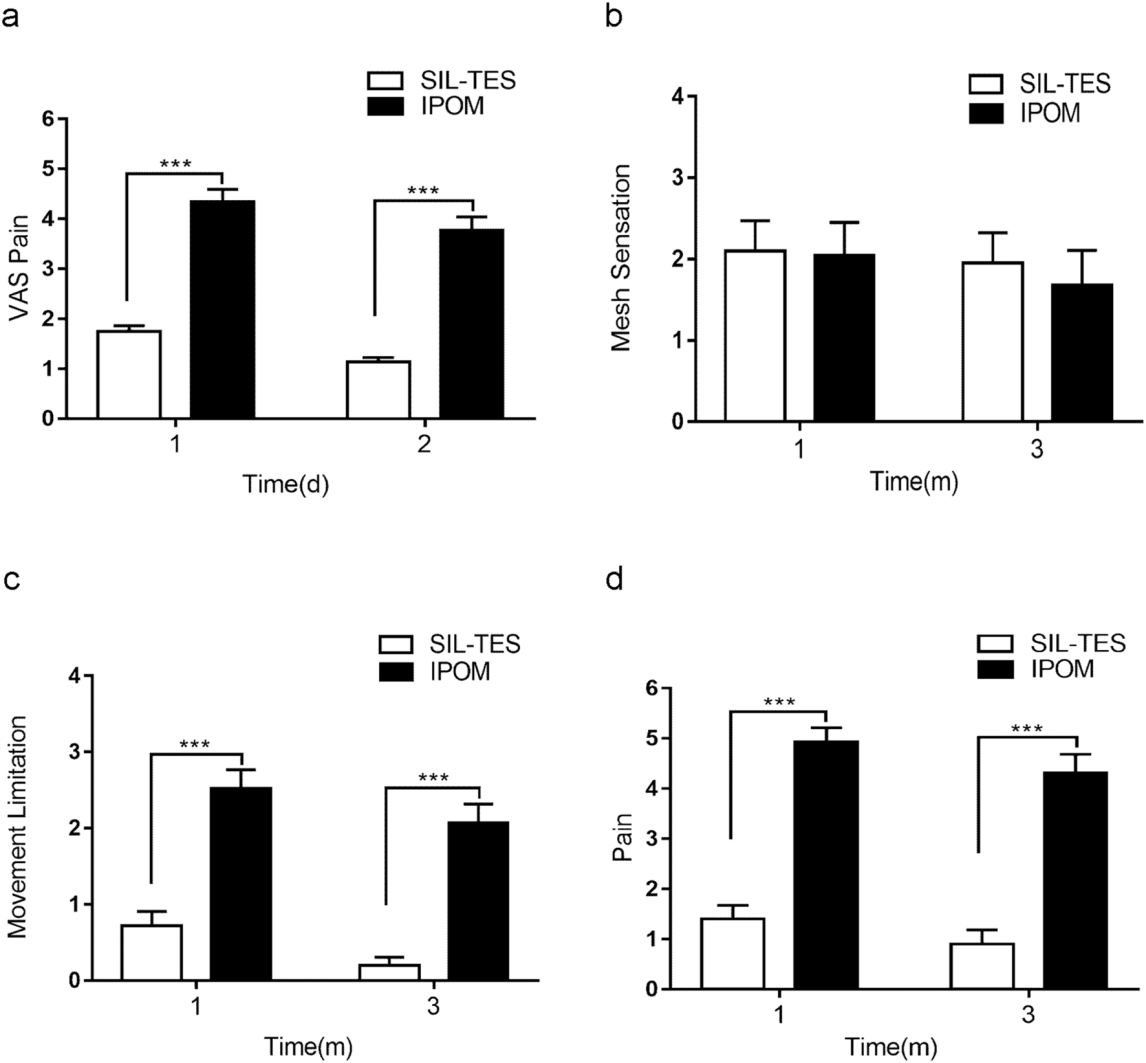
Post-operative pain score assessed by VAS (**a**) and QOL assessed by CCS over time (**b**–**d**)

Pain at 24 and 48 h post procedure was significantly less in the SIL-TES group than in the IPOM group (Figure). Moreover, CCS was used to evaluate QoL at one month and three months after surgery. Mesh sensation score was comparable between the two groups at all postoperative time points (Figure). However, postoperative pain and movement limitation scores were significantly higher in the IPOM group than in the SIL-TES group at 1 month (4.93 ± 0.28 versus 1.75 ± 0.28, *p* < 0.0001 and 2.52 ± 0.24 versus 1.15 ± 0.18, *p* < 0.0001, respectively) and at 3 months (4.32 ± 0.37 versus 0.9 ± 0.29, *p* < 0.0001 and 2.06 ± 0.25 versus 0.69 ± 0.11, *p* < 0.0001, respectively) (Fig. 3).

## Discussion

Recently, ESR for VH has been reported by an increasing number of scholars [3–8, 11, 16–19]. Although this technique is known by several names, including totally endoscopic sub-lay and enhanced-view TEP, at its core, it involves using laparoscopy to mimic open sub-lay repair. TES is a key aspect of this technique, and it seems to be a promising trend. To reduce trauma and improve cosmetic outcomes, our team innovatively combined SILS and TES to treat small and medium VHs. Since Hanh Tran’s 2015 report of seven cases of direct inguinal hernia and the semilunar hernia repaired using SIL-TEP [20], this paper is the largest report on SIL-TES mesh VH repair. It also compares SIL-TES mesh repair and IPOM repair. Analyses of retrospectively collected data of 104 patients from five hernia centers revealed that, in terms of intraoperative complications, postoperative experience, and cost-effectiveness, SIL-TES mesh repair is significantly superior to IPOM repair.

Usually, adhesiolysis is an essential part of IPOM repair. With an incidence rate of up to 11%, inadvertent enterotomy is the most common intraoperative complication of abdominal adhesiolysis [21–23]. It is associated with sepsis, abdominal complications, surgical-site infection, long hospital LoS, and mortality rate of up to 8% [23]. Further, it was reported that the rate of intestinal injury, especially during VH repair, might be higher with the laparoscopic approach than with the open approach [24]. In this study, there were two cases of intraoperative bowel injury and one case of postoperative intestinal obstruction in the IPOM group. However, no such complications occurred in the SIL-TES group, which may be because the TES approach has minimal effect on abdominal viscera. Meanwhile, extensive abdominal adhesiolysis placed more drainage in patients in the IPOM group, and their hospital LoS increased accordingly.

In addition, pain at 24 and 48 h post procedure was significantly greater in the IPOM group than in the SIL-TES group. Post-operative pain and movement limitation scores evaluated using CCS at one month and three months were significantly higher in the IPOM group than in the SIL-TES group. In the TES approach, lightweight polypropylene mesh is sandwiched between muscle and posterior sheath and placed without fixation using tacks. This has two advantages: non-fixation of mesh and cost-effectiveness as the affordable polypropylene mesh is used instead of the expensive composite mesh with an anti-adhesion barrier. This is the main reason the treatment was more cost-effective in the SIL-TES group than in the IPOM group. Furthermore, mesh fixation without tacks helped reduce postoperative pain, and this is consistent with some study reports stating that a direct relationship exists between aggressive mesh fixation and postoperative pain [25, 26]. Meanwhile, SILS circumvents the multi-channel puncture of conventional laparoscopy, resulting in less postoperative pain [27, 28], low risk of damage to abdominal wall vessels during trocar instrumentation, and less impact on the integrity of the abdominal wall [29]. It should be added that although intermittent transabdominal stitches was used to close the defect in 80% of the SIL-TES group as in the IPOM group, there was still a significant difference in closure method between the two groups. This means that transabdominal stitches is one of the major causes of postoperative pain, and that continuous reverse sewing using barbed sutures has the potential to reduce postoperative pain in SIL-TES. All of these contribute to the improvement of the subjective postoperative experience of patients. The results presented above show that SIL-TES mesh repair effectively combines the advantages of the two techniques.

One concern regarding SIL-TES mesh repair is the inline vision and chopsticks effect experienced during SILS; these phenomena increase the operative difficulty for surgeons, especially inexperienced surgeons. This could be the main reason mean operation time was significantly greater in the SIL-TES group than in the IPOM group. In the initial phase of the SIL-TES technique, the triangle layout can be improved by adding one auxiliary channel to reduce operative difficulty due to the chopsticks effect. However, the operation can be simplified in some ways with SILS. Under the visual field of SILS and under laparoscopic guidance, the extra-peritoneal space is accurately and completely established, and this is performed with greater ease and safety during SILS than during routine TES mesh repair. There have been reports of increased incidence of incisional hernia after SILS [30], which may be another concern with SIL-TES mesh repair. But in this study, the mean LoI in the SIL-TES group was 2.2 cm, and there was no incidence of incisional hernia over the mean follow-up duration of 12 months. We believe that exact closure of the fascial layer is effective for preventing incisional hernia, and we intend to confirm this hypothesis by increasing the number of surgical patients and extending the follow-up duration.

This paper has some limitations. First, the sample size of this study is relatively small, and the cases have certain selectivity, so the external validity of the results is relatively limited. As an innovative use of the SILS, our team is increasing the sample size and proportion of complex cases, such as choosing to repair larger defects (≥ 4 cm) or more incisional hernias. Second, the proportion of cases of primary VH with relatively few adhesions [31] was greater in the SIL-TES group than in the IPOM group. Therefore, operative difficulty and, to a certain extent, the rate of postoperative seroma formation was lower in the SIL-TES group than in the IPOM group. These differences affected the results of the comparison between the two groups. Third, this study did not include scar evaluation (i.e., cosmetic outcome evaluation) after SILS. The importance of this report is in the sharing of preliminary experience of combining the SILS and TES techniques of VH repair, and we plan to conduct a prospective randomized controlled study with an expanded sample size in future.

## Conclusion

The concept of “abdominal wall problems back to the abdominal wall” has widely been supported in recent years. For experienced surgeons, the combination of the SILS and TES approaches of VH repair is a favorable complement to routine laparoscopy. This novel method looks to combine the best aspects of the SILS and TES approaches. This study preliminarily shows that the TES technique is safe and effective during SILS. Therefore, we believe that further research data should be obtained through the accumulation of surgical volume to support the development of this novel technique.

## Data Availability

Available if requested.
